# Fingolimod attenuates experimental autoimmune neuritis and contributes to Schwann cell-mediated axonal protection

**DOI:** 10.1186/s12974-017-0864-z

**Published:** 2017-04-26

**Authors:** Björn Ambrosius, Kalliopi Pitarokoili, Lisa Schrewe, Xiomara Pedreiturria, Jeremias Motte, Ralf Gold

**Affiliations:** 1 Department of Neurology, St. Josef Hospital, Ruhr-University, Bochum, Germany; 2 Current address: Department of Neurology, University Hospital Bern, University of Bern, Bern, Switzerland

**Keywords:** Chronic inflammatory demyelinating neuropathy (CIDP), Nerve conduction studies, Nerve excitability, Guillain-Barré syndrome (GBS), Myelin

## Abstract

**Background:**

Fingolimod, a sphingosine-1-phosphate receptor modulator with well-described immunomodulatory properties involving peripheral immune cell trafficking, was the first oral agent approved for the treatment of relapsing remitting multiple sclerosis. Analogous immunomodulatory treatment options for chronic peripheral autoimmune neuropathies are lacking.

**Methods:**

We tested fingolimod in the animal model of experimental autoimmune neuritis in Lewis rat. Six to eight-week-old female rats were immunized with P2 peptide and from this day on treated with fingolimod. Histology of the sciatic nerve was done to analyze T cell and macrophage cell count, intercellular adhesion molecule (ICAM) and amyloid precursor protein (APP) expression, as well as apoptotic Schwann cell counts.

**Results:**

Preventive oral treatment with 0.1 mg/kg up to 3 mg/kg fingolimod once daily dissolved in rapeseed oil completely ameliorated clinical neuritis signs. It reduced circulating peripheral blood T cells and infiltrating T cells and macrophages in the sciatic nerve, whereas at the same time, it preserved blood-nerve barrier impermeability. Most importantly, fingolimod showed beneficial properties on the pathogenic process as indicated by fewer apoptotic Schwann cells and a lower amount of amyloid precursor protein indicative of axonal damage at the peak of disease course.

**Conclusions:**

Taken together, orally administered low-dose fingolimod showed an impressive immunomodulatory effect in the rat model of experimental autoimmune neuritis. Our current observations introduce fingolimod as an attractive treatment option for neuritis patients.

## Background

Every year, many thousands of people worldwide are affected by autoimmune peripheral neuropathies. Guillain-Barré syndrome (GBS) and chronic inflammatory demyelinating polyneuropathy (CIDP) represent the majority of human immune neuropathies [1, 2]. They are characterized by an autoimmune cellular and humoral response against proteins of the myelin sheath and nerve axons [3–5]. Although immunosuppressive and immunomodulatory therapy has improved the therapeutic options treating GBS or CIDP, still 25% of the patients do not respond to first-line treatment adequately and 50% of the patients experience clinical relapses after treatment [3, 6]. This clearly indicates the need to broaden the spectrum of therapeutic options for neuritis patients [7].

The mostly used animal model to mimic acute peripheral neuropathies is the experimental autoimmune neuritis (EAN) in Lewis rats, which is induced by peripheral nervous system (PNS) antigens (e.g., P2) emulsified in complete Freund’s adjuvant (CFA) [8, 9]. EAN mimics many aspects of the humoral and cellular response, electrophysiological characteristics, and histological appearance of GBS and CIDP [10, 11]. In EAN, mostly macrophages and CD4 T cells migrate to the PNS after disease onset and cause nerve damage via direct phagocytic attack, via T cell mediated cytotoxicity or via soluble factors like cytokines and free oxygen radicals [5, 12, 13].

Fingolimod is a sphingosine 1-phosphate receptor (S1PR) modulator and became approved for multiple sclerosis treatment in 2010. In two phase 3 studies, it significantly improved patients relapse rate and decreased risk of disability progression compared to placebo group (FREEDOMS I, FREEDOMS II) [14, 15]. In contrast, in another phase 3 study, fingolimod failed to slow down disease progression in primary progressive multiple sclerosis (INFORMS) [16].

Mainly, fingolimod blocks the egress of lymphocytes from secondary lymphoid organs, which results in a reduced lymphoid cell count in peripheral blood and to less neuroinflammation [17]. More and more findings add up, which suggest that the effect of fingolimod is not limited to reduced neuroinflammation, due to repulsing T cells in lymphatic organs. A neuroprotective effect of fingolimod against glutamatergic excitotoxicity was described in experimental autoimmune encephalomyelitis (EAE) mice [18, 19] and also in a rat model of autism, focussing on changed glutathione levels and superoxide dismutase activity [20].

Studies in EAN have already highlighted that fingolimod, applied intraperitoneally, ameliorates the disease course by significantly decreasing the number of infiltrating macrophages and T cells into peripheral nerves [21]. The same research group showed that the percentage of FoxP3 positive T cells was increased in the peripheral blood and nerves of EAN rats whereas at the same time, IL17^+^ cells were reduced in EAN lesions [22]. Further studies in EAE mice indicate an effect of fingolimod on the PI3K/AKT/mTor pathway in lymphocytes. The outcome of this is in an altered Th1/regulatory T cell differentiation and altered IL17^+^-cells distribution in the central nervous system (CNS) [22, 23].

The efficacy and safety of 0.5 mg up to 1.25 mg fingolimod administered orally once daily versus placebo in 156–200 patients with CIDP has already been a subject of a clinical study (FORCIDP, ClinicalTrials.gov Identifier NCT01625182), but without any PubMed listed publications so far. However, the pathophysiological mechanisms mediating the effect of fingolimod in EAN still remain elusive. Therefore, we focused on the effect of orally administered fingolimod in EAN as a novel immunomodulatory and potentially neuroprotective treatment option for inflammatory neuropathies.

## Methods

### Antigens

Bovine myelin P2 peptide corresponding the amino acids 53–78 (P2_53–78_) was used forimmunization of Lewis rats. The neuritogenic peptide P2_53–78_ was synthesized by Dr. Rudolf Volkmer from Charité University (Berlin, Germany).

### Induction of EAN and assessment of clinical score

Female Lewis rats with an age of 6–8 weeks were purchased from Charles River (Sulzfeld, Germany). Animals were in the range of 160–180 g when immunized and at least 1 week in the animal facility to get accustomed to the new environment. Rats were housed under standardized, pathogen-free conditions at the local animal facility (Medical Faculty, Ruhr-University Bochum, Bochum, Germany) where food and water were given ad libitum.

For immunization, 10 mg/kg xylazine (Xylavet, CP-Pharma, Burgdorf, Germany) and 50 mg/kg ketamine (CP-Pharma, Burgdorf, Germany) were used intraperitoneally (i.p.) to anesthetize the rats. Two hundred fifty microgram P2_53–78_ in phosphate buffer saline (PBS) emulsified in equal volume of CFA containing 1 mg/ml Mycobacterium tuberculosis H37RA (Difco, Detroit, USA) were used to subcutaneously inject into the tail base of the rats. From the day of immunization, animals were weighed and scored daily. To determine a disease score, a tenfold system was used (0 normal; 1 less lively; 2 impaired righting/limb tail; 3 absent righting; 4 ataxic gait, abnormal position; 5 mild paraparesis; 6 moderate paraparesis; 7 severe paraplegia; 8 tetraparesis; 9 moribund; 10 death) (Enders et al. 1998). Animal experiments were approved by the North-Rhine-Westphalia, Germany authorities (Az.: 84-02.04.2015.A420).

### In vivo treatment with fingolimod

Fingolimod was purchased by Sigma-Aldrich (Taufkirchen, Germany) as a >98% pure powder, which was dissolved in rapeseed oil (Fauser Vitaquell, Hamburg, Germany). From the day of immunization, rats were given 200 μl rapeseed oil with different concentrations (0.1–3 mg/kg) of fingolimod by oral gavage till day 17 or day 21 post immunization (p.i.) daily. The rats were randomly divided into different groups (four animals per group). The control group was treated with 200 μl pure rapeseed oil, whereas the active pharmaceutical ingredient groups were treated with different concentrations of fingolimod dissolved in 200 μl rapeseed oil. The tested concentrations of fingolimod ranged from 0.025 mg/kg up to 3 mg/kg fingolimod daily.

### Flow cytometric analyses of immune cells in peripheral blood

Blood was collected before perfusion at disease maximum (day 17 p.i.) under aseptic conditions. Erythrocytes were lysed using ACK buffer (150 mM NH_4_Cl, 10 mM KHCO_3_, 0.1 mM Na_2_EDTA). Cells were washed twice in Dulbecco`s phosphate buffer saline (DPBS) (ThermoFisher, Schwerte, Germany) and stained with monoclonal antibodies for CD4 or CD11b (BD Pharmingen, Heidelberg, Germany). Cells were analyzed in a FACS Canto II (BD Pharmingen, Heidelberg, Germany), and DIVA Software (BD Pharmingen, Heidelberg, Germany) was used for cell population analysis.

### Nerve conduction studies

Nerve conduction tests were performed 1 day before immunization (day 1 p.i.) and at the end of the experiment at day 17 p.i. (maximum disease course) or day 21 p.i. (recovery phase) as described earlier [7].

According to the immunization protocol, 10 mg/kg xylazine and 50 mg/kg ketamine were used i.p. to anesthetize the rats. A fully digital recording Keypoint apparatus (Dante, Skovlunde, Denmark) was used in combination of paired needle electrodes, which were inserted into the sciatic notch (hip, proximal) and into the popliteal fossa (knee, distal) to examine the amplitude and latencies of compound muscle action potentials (CMAPs). CMAPs were used to assess the sciatic nerve motor conduction in rats. The sciatic nerve was stimulated with a pulse of 0.05 ms and motor nerve conduction velocity (MNCV), and F waves were recorded. Temperature differences were minimized by conducting the study as soon as the anesthesia had taken effect and by warming the leg with a heating lamp.

### Histopathological assessment and immunohistochemistry

On disease maximum (day 17 p.i.) or in recovery (day 21 p.i.), animals were sacrificed using CO_2_. Transcardial perfusion with PBS (ThermoFisher, Schwerte, Germany) was done. The right sciatic nerves were dissected and embedded in Neg-50 (ThermoFisher, Schwerte, Germany), snap frozen, and stored at −80 °C for histopathological assessment. The nerves were sectioned (10 μm) on a cryostat (ThermoFisher, Schwerte, Germany) and mounted on deep frozen approved glass slides (Hartenstein, Würzburg, Germany).

Immunohistological staining were performed using the DAKO animal research kit for primary mouse antibodies (Dako, Hamburg, Germany) as described by the manufacturer’s protocol. Monoclonal antibodies against T cells (Pan T Cells, 1:100, Hycultec, Beutelsbach, Germany) and against macrophages (ED1, CD68, 1:100 Hycultec, Beutelsbach, Germany) were used. Stained cells were counted at ×40 magnification for 8 sections per animal. Counts were multiplicated by 16 to archive cells/mm^2^ tissue.

For identification of APP (amyloid precursor protein), ICAM-1 (intercellular adhesion molecule-1), S-100 and Caspase-3 expression, the following antibodies were used: anti-APP (1:100, Merck Millipore, Darmstadt, Germany), anti-ICAM-1 (1:100, Antibodies-online, Aachen, Germany), anti-S-100 antibody (1:100, DAKO, Hamburg, Germany), and anti-Caspase-3 (1:100, Abcam, Cambridge, England). Secondary antibodies conjugated with Alexa 488 and Alexa 555 (1:1000) (ThermoFisher, Schwerte, Germany) were used according to manufacturer’s protocol and DAPI-Fluoromount (4′,6′ diamino-2-phenylindole 2HCl, Biozol, Eching, Germany) was used for fluorescent staining of DNA. Fluorescent signals were detected using an inverted fluorescence microscope (BX51; Olympus, Tokyo, Japan) equipped with an Olympus DP50 digital camera. For assessment of fluorescent staining, images (×20 magnification) of eight transverse sections of the sciatic nerve from each animal were digitally generated (Cell^F 5.1, Olympus, Tokyo, Japan). The percentage of the area of APP or ICAM-1 stained cells or the cell count of co-stained S-100 and caspase-3 cells per section was determined using image analysis software ImageJ (National institutes of Health, Bethesda, USA). Omission of the primary antibodies served as negative control.

### Statistical analysis

Statistical analyses were performed using GraphPad Prism 6 (GraphPad Software Inc., San Diego, USA). Area under the curve (AUC) was calculated for clinical courses and analyzed by one-way analysis of variance (ANOVA) combined with Tukey’s multiple comparison test. Histological, electrophysiological, and flow cytometry experiments were also compared using ANOVA combined with Tukey’s multiple comparison test. Data is presented as mean ± SEM. Probability level (*p* value) are indicated as *p < 0.05, **p < 0.01, and ***p < 0.001.

For histological analysis, slides were blinded by a not-involved third person and labeled with a numeric-code, which was unblinded after analysis. Treated animals were not blinded due to the highly effective treatment with fingolimod.

## Results

### Fingolimod suppresses experimental autoimmune neuritis in rats

**Fig. 1:**
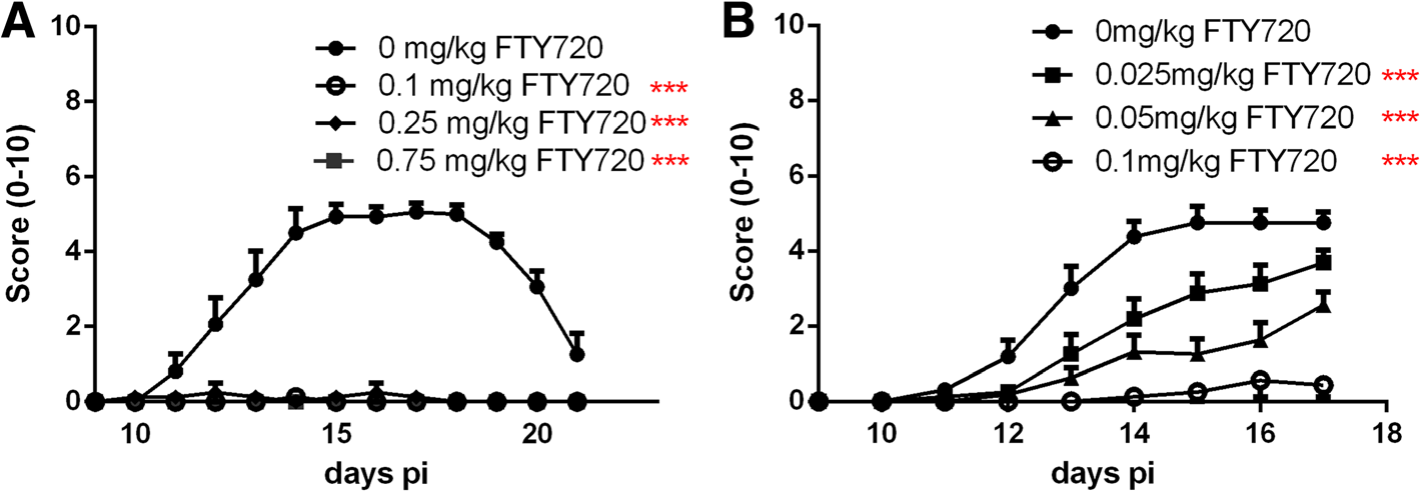
Fingolimod ameliorates disease course in EAN. Clinical score of immunized rats treated with different concentrations of fingolimod until complete decline of symptoms (**a**) or till near maximum disease course (**b**). After induction of EAN on day 0, animals were treated daily and orally with different concentrations of fingolimod. Concentrations of 0.1 mg/kg fingolimod and upwards prevented development of clinical symptoms. One to two independent experiments with four animals per group were performed. Statistical analysis and *p* value were obtained by calculating the area under the curve of each condition. Comparison of AUC was done using one-way ANOVA combined with Tukey’s multiple comparisons test; ***p < 0.001

Rats were immunized with peptide P_53–78_ on day 0. From this day on, daily oral gavage of fingolimod dissolved in rapeseed oil started. Clinical signs of EAN occurred around day 11 p.i. and progressed till day 17, before clinical recovery befall. Treatment of the rats with concentrations of 0.1 mg/kg and more, nearly completely suppressed clinical EAN course and caused significantly less clinical signs compared to control animals (*p* < 0.001, AUC, ANOVA, Tukey’s multiple comparison test) (Fig. 1a). Concentrations of 0.025 mg/kg fingolimod and 0.05 mg/kg fingolimod caused a dose-dependent, but not entire decrease of clinical signs compared to the control group (*p* < 0.001) (Fig. 1b) (2 independent experiments, 4 animals per group). In both conditions, animals treated till recovery phase and till maximum disease course, treatment with 0.1 mg/kg fingolimod resulted in a significant ameliorated disease course (*p* < 0.001). The EAN incidence in the control group was 100%. Higher concentrations up to 3 mg/kg fingolimod maintain the observed effect (data not shown). No significant body weight reduction or further toxic effects of fingolimod at any dosage were found (data not shown). In contrast, therapeutic treatment with fingolimod (0.1, 0.25, and 0.75 mg/kg) did not show any beneficial effect compared to the control group (1 independent experiment, 4 animals per group) (data not shown).

**Fig. 2:**
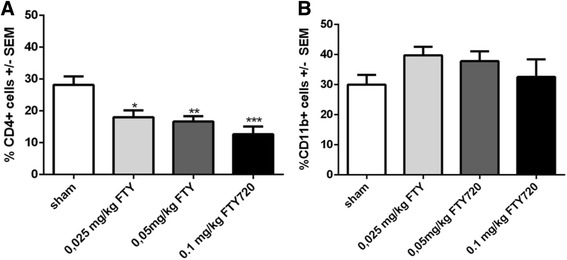
Fingolimod significantly decreases circulating T cells in the blood. FACS analysis of EAN rats treated with different concentrations of fingolimod. Staining of cells with CD4 (**a**) or CD11b (**b**) antibodies revealed a dose-dependent reduction of T cells in the blood, whereas monocytes/macrophages were not altered. Experiments were performed at maximum of disease (day 17 p.i.) in two independent experiments with four animals per group. One-way ANOVA (*p* < 0.0001) combined with Tukey’s multiple comparison test was performed to calculate statistics. *p < 0.05; **p < 0.01; ***p < 0.001

In order to confirm the effectivity of fingolimod, we tested the peripheral blood cells using flow cytometric analysis at maximum disease course (day 17 p.i.). Our results showed a dose-dependent reduction of circulating CD4^+^ T cells compared to sham-treated animals (0.025 mg/kg fingolimod: *p* < 0.05; 0.05 mg/kg fingolimod: *p* < 0.01; 0.1 mg/kg fingolimod: *p* < 0.001) (Fig. 2a). In contrast, CD11b expressing cells were not altered by different concentrations of fingolimod in a significant way (Fig. 2b) (2 independent experiments, 4 animals per group).

### Fingolimod improves proximal and distal nerve conduction

To investigate the potential protective effects of fingolimod against demyelination, we performed electrophysiological measurements of the right sciatic nerve before immunization (day 1 p.i.), at maximum disease course (day 17 p.i.) and in the recovery phase of the animals (day 21 p.i.). MNCV was used as an indicator for demyelination. Also, F wave latencies were used as a marker for lumbar root involvement as described before [7, 24].

**Fig. 3:**
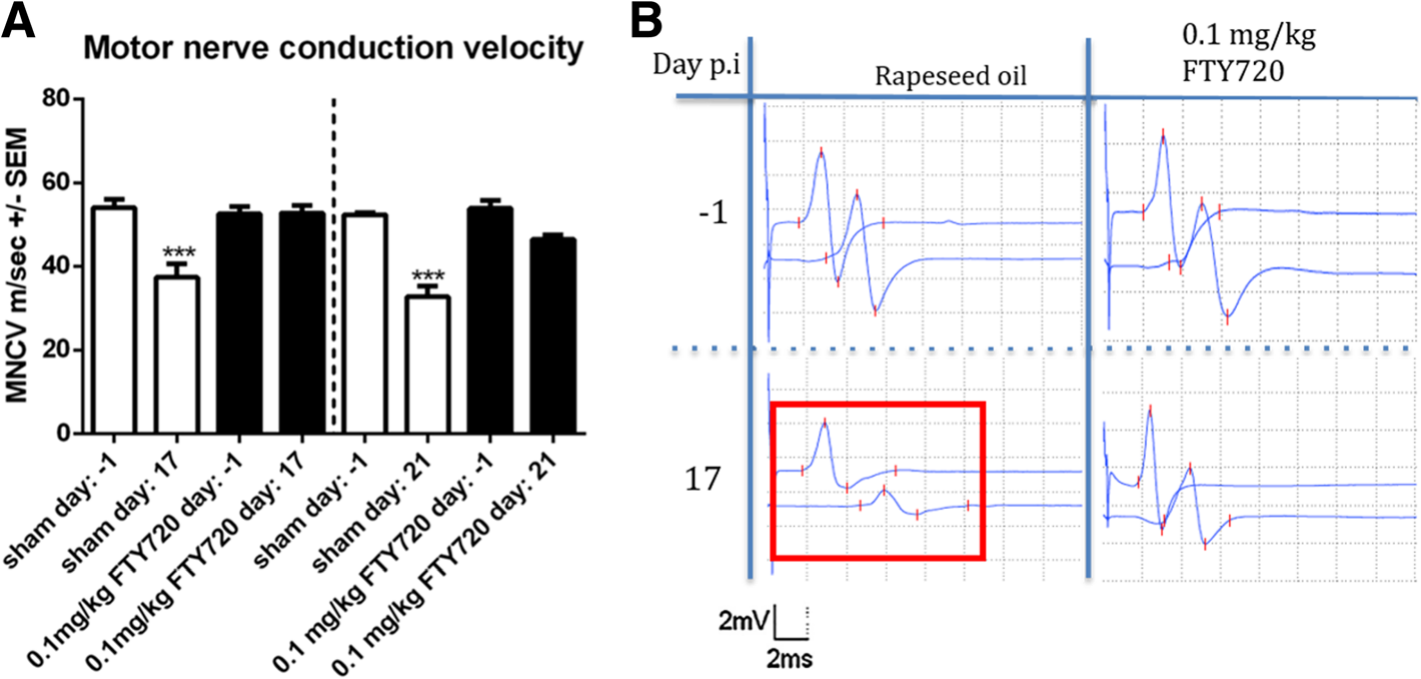
Fingolimod protects against demyelinating damage. Electrophysiological testing of motor nerve conduction velocity (MNCV) (**a**) and example of nerve conduction block in the lower left corner (**b**). Animals were examined 1 day before immunization and at either maximum of the disease (day 17) or in the recovery phase (day 21). One to two independent experiments were performed with four animals per group. MNCV was compared using a one-way ANOVA (*p* < 0.0001) combined with Tukey’s multiple comparison test. ***p < 0.001

Both at the maximum disease course and in the recovery phase, sham-treated animals showed a significant decline in MNCV compared to baseline-testing at day 1 (*p* < 0.001). Treatment with 0.1 mg/kg fingolimod prevented decrease of MNCV compared to baseline-testing at day 1 (Fig. 3a) (1–2 independent experiments, 4 animals per group). These results were also confirmed by higher concentrations of fingolimod which were sufficient to supress electrophysiological signs of demyelination (data not shown).

F wave latency was significantly prolonged in control groups at maximum disease course in comparison to baseline-testing (Fig. 3b).

### Fingolimod decreases infiltrating T cells and macrophages in sciatic nerves

**Fig. 4:**
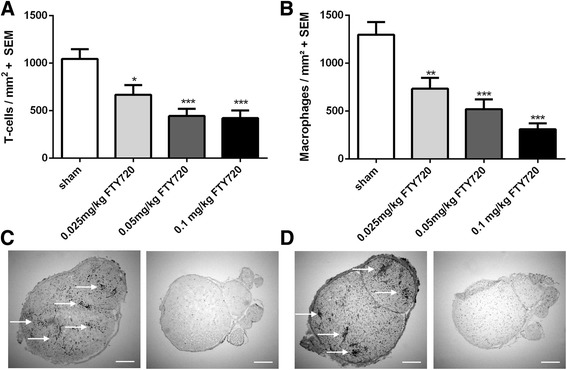
Infiltrating T cells and macrophages in the sciatic nerve are reduced by fingolimod. Pan T cell staining (**a**) and ED1 macrophage staining (**b**) of the sciatic nerve at maximum disease course (day 17). Pictures of the sciatic nerve stained again T cells (**c**) and macrophages (**d**). Left picture displays sham-treated animals and right picture displays 0.1 mg/kg fingolimod-treated animals. Two independent experiments were performed with four animals per group. One-way ANOVA (*p* < 0.0001) combined with Tukey’s multiple comparison test was performed to calculate statistics. *p < 0.05; **p < 0.01; ***p < 0.001

We questioned if the beneficial effect of fingolimod on EAN disease course goes along with a reduced T cell and macrophage cell infiltration in sciatic nerves. We performed T cell and macrophage staining on frozen nerve slides. T cell infiltrates were significantly suppressed by fingolimod concentrations of 0.025 mg/kg (*p* < 0.05), 0.05 mg/kg (*p* < 0.001), and 0.1 mg/kg (*p* < 0.001) at maximum disease course (day 17 p.i.) (Fig. 4 a/c). Related to these results, also infiltrating macrophages showed a significant suppression in the sciatic nerve after treatment with fingolimod concentrations of 0.025 mg/kg (*p* < 0.01), 0.05 mg/kg (*p* < 0.001), and 0.1 mg/kg (*p* < 0.001) at maximum disease course (Fig. 4 b/d) (2 independent experiments, 4 animals per group). For both, T cells and macrophage infiltrates, fingolimod seemed to have a dose-depended reducing impact. In recovery phase, a similar picture of significantly suppressed T cells and macrophages by fingolimod in the sciatic nerve was achieved (data not shown).

**Fig. 5:**
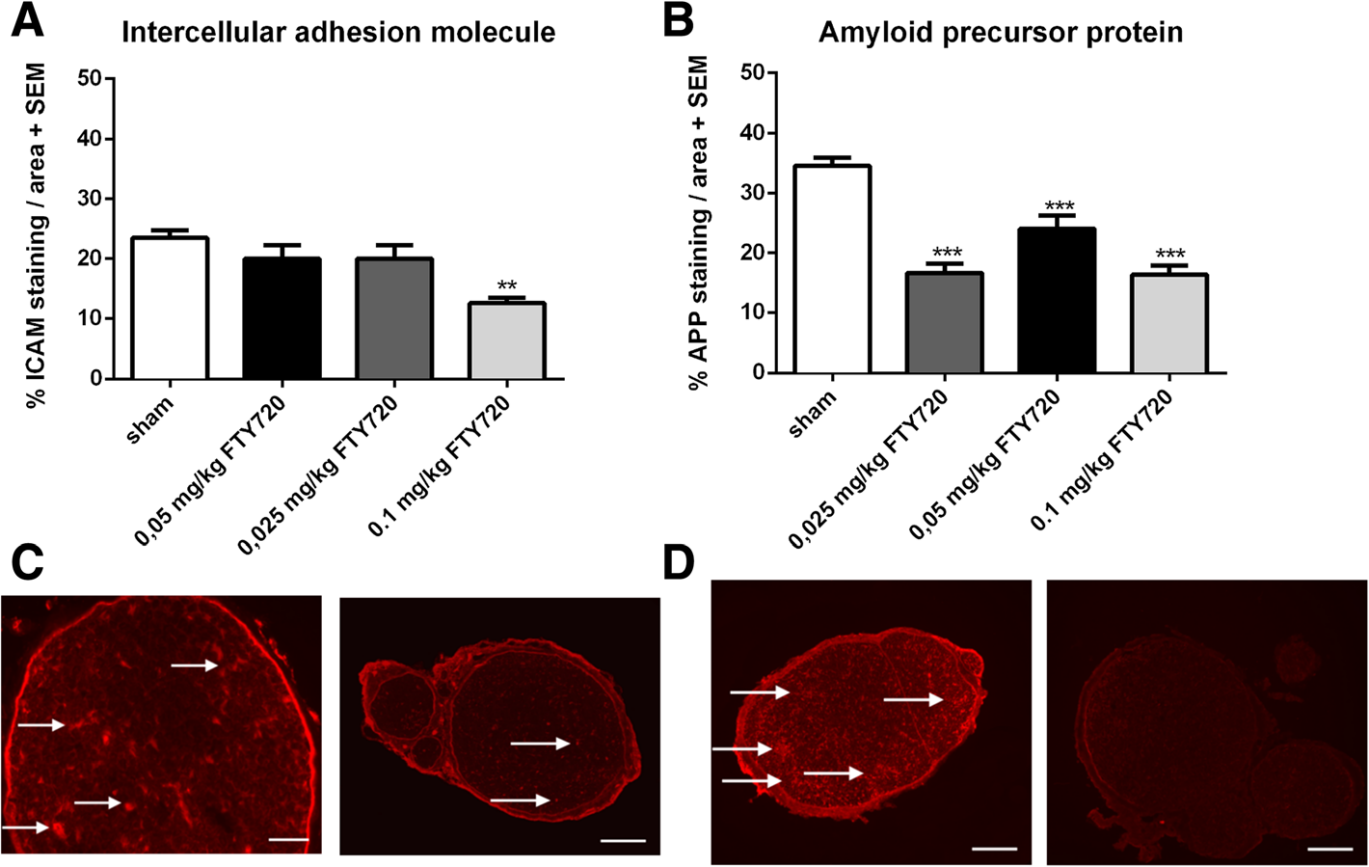
ICAM and APP are reduced by fingolimod. ICAM staining and APP staining of sciatic nerve at maximum disease course (day 17). Percentage of ICAM stained area (**a**) and exemplary pictures of sham-treated animals (×4 magnification, ×40 magnification) (**c**, *left*) and 0.1 mg/kg fingolimod-treated animals (**c**, *right*). Percentage of APP-stained area per slide (**b**) and exemplary pictures of sham-treated animals (**d**, *left*) and 0.1 mg/kg fingolimod-treated animals (**d**, *right*). One independent experiment was performed using four animals per group. One-way ANOVA (*p* < 0.001) and Tukey’s multiple comparison test were performed. **p < 0.01; ***p < 0.001

Additionally, a significant reduction of ICAM-1 expression in the sciatic nerve was only engendered by treatment of the animals with 0.1 mg/kg fingolimod compared to the control group at maximum disease course, whereas lower concentrations of fingolimod had not the ability to significantly lower ICAM-1 expression (day 17 p.i.) (*p* < 0.01) (Fig. 5a/c) (1 independent experiment, 4 animals per group).

### Fingolimod protects peripheral nerves from early axonal damage and reduces Schwann cell apoptosis

**Fig. 6:**
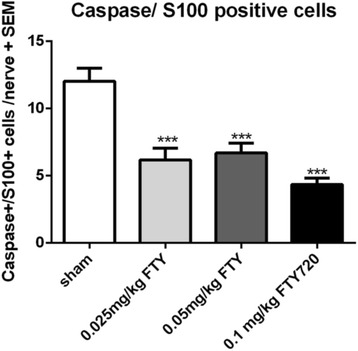
Apoptosis of Schwann cells is reduced by fingolimod treatment. Caspase-3 and S100 co-staining against apoptotic Schwann cells in sciatic nerve at maximum disease course (day 18). A number of double-positive cells were counted per slide. Two independent experiments performed with four animals per group. One-way ANOVA (*p* < 0.0001) combined with Tukey’s multiple comparison test was performed to calculate statistics. ***p < 0.001

The potential direct or indirect neuroprotective effects of fingolimod have been increasingly discussed in the literature in the last years but available data in EAN are lacking. We proceeded to investigate the effect of fingolimod on early axonal damage. A staining of APP in peripheral nerves revealed a reduced expression for fingolimod treated animals. Animals treated with 0.025, 0.05, and 0.1 mg/kg fingolimod showed a reduced expression of APP in the sciatic nerve (*p* < 0.001) compared to the sham-treated animals (Fig. 5b/d) (1 independent experiment, 4 animals per group). A double-staining for apoptotic (caspase-3) Schwann cells (S100) in the sciatic nerve revealed a beneficial effect of fingolimod. There were significantly less apoptotic Schwann cells stained in the sciatic nerves of 0.025, 0.05, and 0.1 mg/kg fingolimod-treated animals compared to sham-treated animals (*p* < 0.001) (Fig. 6) (2 independent experiments, 4 animals per group).

## Discussion

The potential role of fingolimod as a treatment option in the inflamed central nervous system has been already recognized. Research data on potential mechanisms of action of fingolimod mostly stem from animal and human studies in the context of CNS disease as multiple sclerosis [25].

Our experiments reveal an impressive, preventive effect of orally administered fingolimod dissolved in rapeseed oil causing amelioration of clinical EAN signs at very low concentrations of 0.1 mg/kg. Direct comparison of concentrations used in animal models with concentrations known from human studies (0.5–1.25 mg/kg [14]) is difficult. To compare treatment in rats and humans, consideration of body surface area has to be taken into account. Because of that, multiplying the used animal dosage with the conversion factor of 0.162 (summarized in [26]) is necessary and results in a dosage of 0.0162 mg/kg per day compared to 0.0178 mg/kg per day (1.25 mg fingolimod in a random 70 kg patient). In contrast to other studies in EAN [21] and EAE [27], in which higher concentrations of fingolimod between 0.5 and 1 mg/kg were used, we were able to show a significant effect of fingolimod in a 5–10 times decreased concentration, which is comparable to the concentration used for human treatment. Mentionable, only preventive treatment with fingolimod lead to a significant decline of clinical EAN signs, whereas therapeutic treatment with fingolimod failed to achieve a significant beneficial effect compared to immunized, but untreated animals. This clarifies the importance of early treatment with fingolimod to amongst others prevent immune cells from invading peripheral nerves. Our results are in line with former experiments of Zhang et al., where this group tested fingolimod dissolved in PBS administered intraperitoneally at a ten times higher concentration of 1 mg/kg [21]. The clinical effect in our model correlated with a preservation of electrophysiological parameters showing a protection against proximal and distal demyelination. Histologically, we could show the reduction of T cells and macrophages infiltrates in the sciatic nerve during oral application of fingolimod as reported after intraperitoneal application by Zhang et al. [22].

Apart from introducing the immunomodulatory potential of oral fingolimod in EAN for the first time, our results indicate an at least indirect neuroprotective effect in the peripheral nervous system. Amyloid precursor protein histological expression was reduced at the maximum of disease, and most importantly, Schwann cell apoptosis was clearly reduced under fingolimod treatment. It is conceivable, that the reduction of inflammation due to fingolimod treatment lead to an indirect effect, as less Schwann cells undergo apoptosis, and therefore, more Schwann cells support nerves and myelin sheets, which result in a better nerve survival. Also, Schwan cells are crucial for myelin clearance and re-myelination in case of axonal injury [28].

The question posed in order to explain our results is how fingolimod achieved a better Schwann cell survival. On the one hand, as stated before, a decreased inflammatory environment could be responsible for a better Schwann cell survival, but on the other hand, also a direct effect of fingolimod in the PNS is possible. The presence of almost all S1PR on Schwann cells and on axons could imply an effect of the lipophilic fingolimod in the PNS after crossing the blood-nerve barrier [29]. A similar local neuroprotective effect in the central nervous system has been considered in multiple sclerosis patients and experimental autoimmune encephalomyelitis [30].

Yet, our in vivo data do not agree with in vitro data on Schwann cell cultures from Koehne at al. In this study, Schwann cells were treated in vitro with different doses of the active fingolimod metabolite (FTY720P, 10nM, 100nM, and 1 μM), whereas the highest dose induced Schwann cells apoptosis [29]. Clearly, this discrepancy could point out the influence of in vivo experimental environment on immunological happenings. Nevertheless, we can exclude a toxic effect of fingolimod on Schwann cells in our experimental setting.

Another aspect of the local PNS effect of fingolimod is the presence of S1PR also on infiltrating inflammatory cells. A disruption of the balance of S1PR on immune cells could alter proinflammatory cytokine secretion or induce regulatory T cell populations in the PNS, which indirectly increase Schwann cell survival [31].

The local effects of fingolimod in the PNS need to be further investigated, possibly through in vitro studies of Schwann cell—dorsal root ganglia co-cultures or in vivo analyses of cell cycle proteins expression. These experiments are also crucial to understand if the beneficial properties of fingolimod occur as a causality of decreased inflammation or if fingolimod has an active neuroprotective effect in the setting of EAN. These effects are of high therapeutic relevance as a great variety of selective S1PR modulators are currently under development, which may show a specific effect in the PNS.

Another aspect of the local effect of fingolimod in the PNS is its influence on blood-nerve barrier permeability. ICAM-1 expression in sciatic nerve, as a marker for blood-nerve barrier permeability [32, 33], was reduced in 0.1 mg/kg fingolimod-treated rats. T cells and macrophages express ICAM-1 on their surface [34, 35] which could explain reduced ICAM-1 due to less infiltrating cells after fingolimod treatment. Compared to the reduction rates of T cells and macrophages, all concentrations of fingolimod caused a decrease of infiltrating cells, whereas only 0.1 mg/kg fingolimod caused an isolated decreased expression of ICAM-1, arguing for a distinct effect of fingolimod on ICAM-1 expression in the sciatic nerve. This could allude to a differentiated effect of fingolimod on the endothelia cells of the blood-nerve barrier (BNB), which express S1PR [36].

## Conclusions

Concluding the discussion about the pathophysiological mechanisms of fingolimod in EAN, we have to point out that the well-known mechanisms on immune cell trafficking played a crucial role in our model [37]. Fingolimod induced, as expected, reduced T cells in peripheral blood at maximum disease course. This is in line with results in patients with multiple sclerosis, where 0.5 mg/day fingolimod significantly reduce peripheral blood lymphocytes [14]. However, the effects of fingolimod on autoimmune diseases involve an increasing spectrum of immunomodulatory functions, which need to be better understood.

In the current study, we showed that orally administered fingolimod, in a concentration, which can be compared to the therapeutic concentration used in human, is highly effective in EAN and that its therapeutic potential is not restricted to the influence on immune cells in peripheral lymphoid organs. The obtained data points out the importance of further research on this area for patients with autoimmune neuropathies.

## Abbreviations

APP: Amyloid precursor protein
AUC: Area under the curve
Az: Aktenzeichen
BNB: Blood-nerve barrier
CFA: Completes Freud`s adjuvant
CIDP: Chronic inflammatory demyelinating polyneuropathies
CMAP: Compound muscle potentials
DAPI: 4′,6-Diamidin-2-phenylindol
DPBS: Dulbecco’s phosphate buffer saline
EAE: Experimental autoimmune encephalomyelitis
EAN: Experimental autoimmune neuritis
GBS: Guillain-Barré syndrome
i.p.: Intraperitoneally
ICAM1: Intercellular adhesion molecule 1
MNCV: Motor nerve conduction velocity
p.i.: Post immunization
PBS: Phosphate buffer saline
PNS: Peripheral nervous system
*p* value: Probability level
S1PR: Sphingosine-1-phosphate receptor
